# Creativity in the South Korean Workplace: Procedural Justice, Abusive Supervision, and Competence

**DOI:** 10.3390/ijerph19010500

**Published:** 2022-01-03

**Authors:** Woo-Sung Choi, Seung-Wan Kang, Suk Bong Choi

**Affiliations:** 1 Seoul School of Integrated Sciences & Technologies, 46 Ewhayeodae 2-gil, Fintower, Sinchon-ro, Seodaemun-gu, Seoul 03767, Korea; choikhan_ws@stud.assist.ac.kr; 2 College of Business, Gachon University, 1342 Seongnamdaero, Sujeong-gu, Seongnam 13120, Korea; 3 College of Global Business, Korea University, 2511 Sejong-ro, Sejong City 30019, Korea

**Keywords:** creativity, procedural justice, abusive supervision, competence, conservation of resource theory

## Abstract

Innovation is now a feature of daily life. In a rapidly changing market environment and amid fierce competition, organizations pursue survival and growth through innovation, and the key driver of innovation is the creativity of employees. Because the value of creativity has been emphasized, many organizations are looking for effective ways to encourage employees to be creative at work. From a resource perspective, creativity at work can be viewed as a high-intensity job demand, and organizations should encourage it by providing and managing employee resources. This study is an attempt to empirically investigate how competence and abusive supervision affect the relationship between procedural justice and creativity from the conservation of resources perspective. Findings from two-wave time-lagged survey data from 377 South Korean employees indicate that procedural justice increases creativity through the mediation of competence. Furthermore, abusive supervision has a negative moderating effect on the relationship between procedural justice and competence. The findings show that competence moderates the relationship between procedural justice and creativity and that the lower the level of abusive supervision, the greater the effect of procedural justice on competence and creativity.

## 1. Introduction

In the face of fierce global competition and rapid environmental change, organizational competitiveness depends on the creativity of employees [1]. Many scholars find that innovative behavior has a significant impact on organizational performance and that creativity is the key driver of innovative behavior [2,3,4,5,6]. In other words, creativity is a source of competitive advantage and a basic premise for organizational survival [7,8].

The emerging knowledge economy and technological interventions are changing the existing job profiles, hence the need for different skillsets and technological capabilities [9]. The creativity of members and the organization is essential in acquiring and using these abilities anew. With all business activities supported by AI, autonomy, decentralization, and networking have emerged as important keywords. As business activities, especially for innovation teams, are increasingly organized as autonomous multidisciplinary units requiring creativity, the classical style of order and command will become less and less relevant.

This situation applies to both for-profit and nonprofit organizations (e.g., public health organizations). In this environment, creativity is a major research topic receiving much attention in public health research. For instance, Locke et al. (2019) found that promoting creativity and innovation in government public health agencies can drive a change in government health agencies [10]. It also improves worker satisfaction and workplace environments in government health agencies. In previous studies, innovation based on creativity in public health services improved public health, and innovative actions related to creativity increased the work productivity of healthcare workers [11,12].

One-way individual creativity manifests itself is as suggesting and testing original ideas and applying the knowledge learned to other areas [13]. Individual creativity consists of three components: creative thinking skills, expertise, and motivation. Creative thinking entails recognizing a problem and solving it, and professional knowledge is specific knowledge that can create new ideas [14]. Creativity promotes individual work performance as well as organizational innovation and efficiency, and it is a core competency required in various work areas [5,15,16]. Organizations that do not effectively encourage their employees to be creative can therefore face difficulty remaining competitive.

Creativity is known to be affected by various antecedent factors such as individual characteristics, intelligence, knowledge or technical ability, organizational climate, and leadership style [17,18,19,20,21]. Recently, as the importance of creativity and the need to encourage creativity in organizations have been increasingly highlighted, studies explaining the mechanism from the conservation of resources (COR) perspective are increasing. Researchers have demonstrated the effect of emotional competence on creativity from the point of view of COR theory [22] and shown that atmospheres of psychological safety affect creativity through organization-based self-evaluation [23]. Researchers have also studied various antecedent factors for creativity within the COR framework through studies on the relationships between stress and creativity and between hospitality and creativity [24,25]. In contrast, there has been little research on the structural relationships between variables such as procedural justice, an organizational variable that is likely to affect individual competence and creativity, and abusive supervision, a negative leadership style that can exhaust job resources. Therefore, it is necessary to study this relationship in more detail.

From a COR perspective, creativity can be viewed as a high-intensity job demand. Job demand-resources theory, which extends the COR perspective, presents a model that can explain the phenomenon in various job performance situations. Even under job demands that require sustained physical and mental effort, employees experience high motivation if they have sufficient job resources to handle those demands. When workers receive excessive job demands without adequate job resources, they experience negative mental and physical burdens which, in turn, cause job stress [26].

High-level job demands deplete job resources, which in turn negatively affects employees’ mental health and psychological well-being. When workers are subjected to excessive job demands without adequate job resources, they experience negative mental and physical burdens, leading to job stress [26]. Providing job resources that can respond to high-level job demands is essential for the mental health of employees and a healthy work environment. Boudrias et al. (2011) suggested that job resources significantly impact psychological health [27]. In the current environment where individual innovation and creativity are required for the organization’s survival, clarifying antecedent variables as necessary job resources or inhibiting mechanisms and antecedents affecting creativity are directly related to the employee’s psychological health.

Given the current significance of creativity to organizational success and competitiveness, it is necessary to provide employees with sufficient working resources to incorporate creativity into their work. Among such resources are procedural fairness, an important organizational resource, and competence, generally an essential individual-level job resource. Competence can influence creativity by reflecting an individual’s perceptions of social and organizational resources.

One of the leading factors in the history of creativity research has been the behavior of leaders [16,28,29,30], that is, the intentions, directions, and actions of the people who hold authority within an organization; organizational leadership style has a considerable influence on the creativity of the members. In particular, abusive supervision increases employee turnover, stress, and work-family discord [31], decreases self-confidence and negatively affects reputations [32], and lowers employees’ enthusiasm for work [33]. Therefore, it can be expected that negative supervision styles will have negative effects on individual competence and creativity.

As we have seen so far, creativity leads to organizational innovation and is related to organizational performance. The more employees feel they are involved in the organization’s decision-making process, the more competent they feel, which is the basis for creativity [34,35]. In addition, abusive supervision is expected to have a negative effect on creativity. According to Hobfoll, all types of job resources, such as social and organizational resources, are accumulated and used as needed in situations where job demands are high [36]. As argued by job demand-resource theory, motivation has a positive effect on job performance. Organizations must therefore ensure that their employees accumulate a variety of job resources and use them to unleash their creativity. For this study, we reviewed the literature and applied a conservation of resources perspective to investigate the effects of procedural justice and competence, which are considered essential job resources, and abusive supervision, which is presumed to exhaust job resources, on employee creativity.

## 2. Theoretical Background and Hypotheses

### 2.1. Procedural Justice, Creativity, and COR Theory

According to Hobfoll [37], people tend to maintain and preserve as many resources as possible, but this tendency can lead to stress, job dissatisfaction, and extreme sense of loss when they perceive or experience potential or actual loss of resources. Thus, people want to minimize cognitive or actual resource loss and endeavor to recover as much of those lost resources as they can.

Procedural justice is a concept that researchers have applied to the fairness of procedural decision-making regarding compensation distribution [38,39,40]. However, previous researchers have argued for viewing procedural fairness as a comprehensive concept that not only refers to distributing compensation but includes the fairness of the overall decision-making process within the organization [41,42]. It is known that perceptions of organizational fairness tend to make employees feel obligated to repay the organization’s goodwill by taking actions that will benefit the organization [43]. In addition, several previous researchers have revealed that procedural fairness has a positive effect on work attitudes, performance, and organizational citizenship behavior [44,45].

Under COR theory, procedural justice should have a positive effect on effective job performance as an important job resource that organizations can provide. Empirical researchers on resource conservation theory have found that procedural justice has positive relationships with job performance, psychological possession, and organizational immanence, and is also related to creative behavior [46,47,48]. Based on the above, we proposed the following hypothesis:

**Hypothesis** **1.** *Procedural justice is positively related to creativity.*

### 2.2. Mediating Role of Competence

Competence referred to in this study is self-efficacy in job situations [49]. The concept of competence can be said to be an individual’s intrinsic cognitive abilities that, when interpreted in broad terms, can be generally useful in areas of life [50]. However, Spreitzer (1995) defines competence as a belief in one’s ability to perform tasks as a cognitive component of psychological empowerment [49]. This is the same concept as self-efficacy, in which individuals positively recognize their abilities when performing tasks [51] from the motivational viewpoint of empowerment, and it is not ’general efficacy’ but it can be said to be a concept focused on specialized efficacy, that is, job self-efficacy. 

Competence refers to the desire to exert one’s abilities and act effectively in one’s environment [52]. Competence needs are met when people are given opportunities to interact continuously and effectively with the social environment because competence is not acquired but rather is what makes an individual feel confident and effective through behavior [53]. In other words, responding appropriately in a challenging environment leads to a sense of competence, which in turn promotes a sense of efficacy, which in turn leads to intrinsic motivation. As such, competence and self-efficacy are similar concepts in that they are acquired in a specific area and become tools to successfully accomplish a desired goal [54]. Therefore, previous researchers on self-determination motivation theory incorporated the concept of self-efficacy into their work and defined it as individuals’ judgments of their ability to organize and perform actions necessary to achieve a certain result [55,56].

Competence as self-efficacy in job situations is an individual’s belief in his or her ability to organize and perform the actions necessary to achieve a desired result [57]. It refers to an individual’s beliefs, motivational abilities, cognitive resources, and factors necessary to successfully perform a particular task in a given situation [58]. People with a high sense of competence who face difficult problems attribute the problems to a lack of effort and strive to improve their capacities. Competence helps individuals persevere in difficult situations and promotes challenging responses, which stimulate job performance. Conversely, people with low competence perceive that their abilities are insufficient to achieve their goals, so they avoid tasks or give up even in situations where task achievement is easy [59,60].

As described above, competence is a judgment of whether one can successfully perform a given task, and it means confidence in one’s control and utilization of factors such as knowledge and skills necessary for task performance [61]. Highly competent employees take a proactive approach to difficult job requirements, and therefore competence is highly likely to affect job behavior by reflecting an individual’s perception of social and organizational resources. In other words, employees with a sense of competence will more actively accept when job resources such as procedural justice are provided [62]. In addition, highly competent individuals tend to be highly creative because they have the confidence, knowledge, and skills to generate ideas and apply them in their work, and they tend to challenge and solve uncertainty [63,64].

Previous researchers have determined that procedural justice is an important antecedent of competence [65], and with regard to the subject of this study, competence is a mediator in the relationship between procedural justice and creativity. Researchers have also found competence to be positively related to creativity. Therefore, we can predict a positive relationship between procedural justice and competence and infer its mediating role in the relationship between procedural justice and creativity, and we propose the following hypothesis:

**Hypothesis** **2.** *Competence mediates the relationship between procedural justice and creativity such that procedural justice increases employees’ competence, and the increased competence promotes employees’ creativity.*

### 2.3. Moderating Role of Abusive Supervision

Tepper (2000) defined abusive supervision as the degree to which subordinates perceive persistent aggressive behavior through verbal and nonverbal behavior rather than physical contact [31]. Because members of the organization downgrade their performance when they feel they are being treated adversely, when they experience impersonal supervision from their superiors, they naturally decrease any efforts to help their organizations achieve results [66]. Studies on the negative effects of abusive supervision on organizational members have been steadily increasing. Abusive supervision not only increases employee turnover, stress, and work-family conflicts but also lowers self-confidence, tarnishes reputations, and lowers enthusiasm. It can also have negative effects on employees’ own physical health, such as through serious drinking problems [67], or on the supervisors themselves in the form of physical attacks from employees [68]. In short, abusive supervision has a negative relationship with concepts that have positive effects on organizations [69], and under COR theory, abusive supervision depletes job resources.

Employees seek to recover actual or potential resource losses from a variety of sources, such as from superiors and friends as well as organizations to help rebuild both their resources and their motivation [70,71]. Procedural justice based on organizational support is an important job resource that affects competence, whereas abusive supervision exhausts job resources. Therefore, the interaction between procedural justice and abusive supervision, which are job resources, is expected to affect competence according to the following hypothesis:

**Hypothesis** **3.** *Abusive supervision moderates the relationship between procedural justice and competence such that procedural justice has less impact when abusive supervision is high rather than low.*

### 2.4. Integrated Model: Moderated Mediation Effect

**Figure 1:**
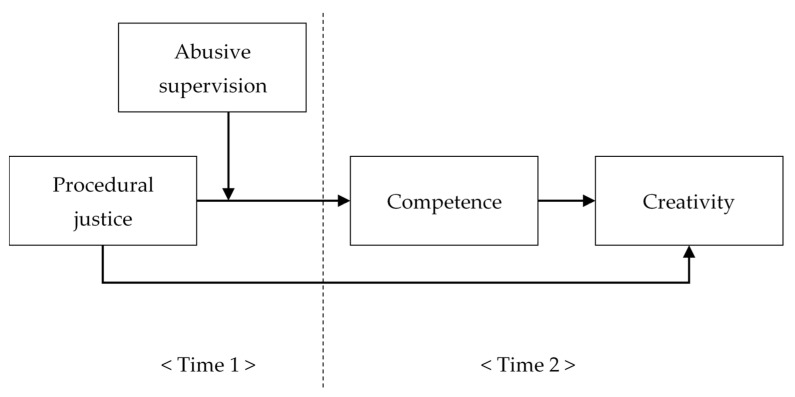
The theoretical research model.

Summarizing the above hypothesis, procedural justice leads to increased competence and thus creativity, and abusive supervision can play a moderating role in this process. Previous researchers have empirically established that abusive supervision regulates the relationship between employees’ internal motivation and organizational creativity, and it also regulates the relationship between employees’ creative processes and organizational creativity by reducing emotional immersion or autonomy [72,73,74]. Therefore, we expect that abusive supervision has a moderated mediating effect in the overall influence of procedural justice on creativity. Meanwhile, decreased competence, a parameter in the relationship between procedural justice and creativity, weakens the positive effect of procedural justice on creativity, and conversely, under low abusive supervision, the influence of procedural fairness increases, effectively increasing competence and its influence on creativity. We model these relationships in the following hypothesis. The theoretical model of this study is depicted in Figure 1.

**Hypothesis** **4.** *The mediation effect of competence on the relationship between procedural justice and creativity will vary such that high abusive supervision will weaken the mediation effect of competence on the relationship between procedural justice and creativity.*

## 3. Methodology

### 3.1. Sample

By dividing the study variables with a four-week time lag and surveying in two rounds, we lowered the possibility of common method bias that can occur with cross-sectional surveys [75]. Prior research indicated that four weeks was long enough to allow for changes in employee psychological factors such as strain but short enough to allow for stability in one’s environment [76,77]. We gathered the target population for this study from the Korean branch of a reputable online survey company with 45 offices in 16 countries worldwide that specializes in academic research. The survey’s target population was drawn at random from an online panel of office workers with bosses working for South Korean companies. Before completing the questionnaires, the participants were informed about the research’s purpose and procedures. They were also informed of their right to withdraw from the survey at any time, as well as the potential benefits and drawbacks of participating; they were then asked to sign an informed consent form. Only those who signed the consent form had their information collected.

The first survey was sent to 500 people via email, and 420 responses were obtained, excluding unreliable responses. The second survey was sent via email to respondents who completed the first survey one month later. Excluding untrustworthy responses (including incomplete responses), we collected and analyzed 337 responses (response rate: 67.4%). Appendix A describes the sampling procedure. The demographics of the respondents were as follows: Men made up 52.8% of the total, while women made up 47.2%. The respondents’ mean age was 41.8 years (SD = 10.33). Bachelor’s and master’s degrees (56.3%) were the most common, followed by junior college degrees (28.5%), high school diplomas (13.4%), and doctorates (1.8%). In terms of their positions, 18.1% were directors and executives in supervisory roles, while 81.9% were not. The average tenure at an organization was 7.9 years (SD = 7.4).

### 3.2. Measures

The survey respondents rated the questionnaire items for the research variables on a five-point Likert scale (with scores ranging from 1 = strongly disagree to 5 = strongly agree). Because the original measurement items were in English, they were translated into Korean and reviewed and corrected by experts. The Korean questionnaire was then translated back into English, and its validity was confirmed through back translation, in which the similarity of linguistic structure and meaning was compared to the original text [78]. Appendix B contains a comprehensive questionnaire.

#### 3.2.1. Procedural Justice

We used the four items developed by Parker et al. [79] to assess procedural justice. Among the questionnaire items were the following: “People involved in implementing decisions have a say in making the decisions,” and “Decisions are made on the basis of research, data, and technical criteria, as opposed to political concerns.” Cronbach’s alpha was 0.79.

#### 3.2.2. Abusive Supervision

We used the five items shortened version of abusive supervision adopted by Mitchell and Ambrose [80] to assess abusive supervision. Among the questionnaire items were the following: “Ridicules me,” and “Makes negative comments about me to others.” Cronbach’s alpha was 0.94.

#### 3.2.3. Competence

We used the three items developed by Spreitzer [49] to assess competence. Among the questionnaire items were the following: “I am self-assured about my capabilities to perform my work activities,” and “I have mastered the skills necessary for my job.” Cronbach’s alpha was 0.85.

#### 3.2.4. Creativity

We used the four items developed by Sung and Choi [81] to assess creativity. Among the questionnaire items were the following: “I supplied new ideas and differing perspectives” and “I combined ideas from different modules and came up with a more integrated view of the phenomena.” Cronbach’s alpha was 0.87.

#### 3.2.5. Control Variables

We used gender, age, education level, position, and organizational tenure as control variables in this study to more clearly confirm the relationships between the variables in the research model. These were chosen based on previous research related to the research variables [82].

### 3.3. Analytical Method

We used STATA 17.0 to perform confirmatory factor analysis (CFA) to determine the model’s validity and hierarchical regression analysis to test our research hypotheses. We used bootstrapping to test the mediation hypothesis as recommended by Preacher and Hayes [83], and we examined the moderated mediation hypothesis by computing Hayes’s [84] index of moderated mediation.

## 4. Result

The means, standard deviations, correlations, and reliability coefficients of the variables are presented in Table 1.

### 4.1. Validity and Common Method Bias Checks

As seen in Table 2, we performed CFA to test the construct validity of study variables. The normed chi-square (*χ*2/*df*) was 1.37 (*χ*2 = 216.78, *df* = 158), which was less than the cutoff value of 3.00 [85]. The comparative fit index (CFI) was 0.98, and the Tucker–Lewis index (TLI) was 0.98, which exceeded the standard cutoff of 0.95 [85]. In addition, the root mean square error of approximation (RMSEA) was 0.03, which was less than the standard cutoff of 0.08 and even less than 0.05, which is a more desirable standard [85]. All the CFA indicators satisfied the standards verification, which we used to determine that our hypothesized measurement model was appropriate for the data. Additionally, we compared the fit of this four-factor model with three competing models and found that the fit was significantly better with the four-factor model. In addition, the average variances extracted (AVEs) and composite reliability (CR) values for all variables satisfied the criteria (AVE > 0.5, CR > 0.7), and the correlations for each construct were lower than the square root of AVE [86,87]. Furthermore, all standardized factor loadings on predicted constructs were above the cutoff of 0.50 [88,89].

We used a two-wave time-lagged survey in this study to minimize the possibility of common method bias, but all variables were measured from employees’ responses. As such, we performed Harman’s single-factor test, and the results showed that the explanatory covariate of the first factor was 25.89%, which indicated that the research data did not suffer from the serious issue of common method variance [75,90].

### 4.2. Hypothesis Testing

To test Hypotheses 1 and 3, we implemented hierarchical multiple regression analyzes, and we tested Hypotheses 2 and 4 with bootstrapping [66,67]. As shown in Model 5 of Table 3, procedural justice was significantly positively related to creativity (*β* = 0.20, *p* < 0.001), and the explanatory power of Model 5 was significantly higher than that of Model 4 (Model 4 → Model 5: ΔR^2^ = 0.04, ΔF = 13.90, *p* < 0.001). Therefore, we determined that Hypothesis 1 was supported; see Table 3.

Hypothesis 2 predicted that competence would mediate the relationship between procedural justice and creativity. As shown in Table 4, the bootstrapping analysis results that did not rely on the normal sampling distribution assumption showed a coefficient of 0.10. The 95% confidence interval (CI) with 10,000 bootstrapped samples did not include zero (0.04, 0.15). Therefore, we determined that Hypothesis 2 was supported.

**Figure 2:**
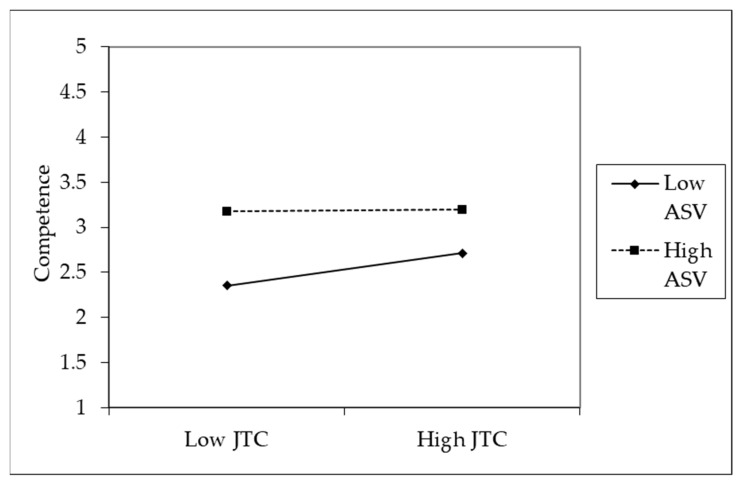
The moderating effect of abusive supervision level on the relationship between procedural justice and competence. JTC = procedural justice, ASV = abusive supervision.

Hypothesis 3 predicted that abusive supervision would moderate the relationship between procedural justice and competence. As shown in Model 3 of Table 3, competence was significantly negatively related to the interaction of procedural justice and abusive supervision (*β* = −0.14, *p* < 0.05), and the explanatory power of Model 3 was significantly higher than that of Model 2 (Model 2 → Model 3: Δ *R*^2^ = 0.04, Δ*F* = 6.73, *p* < 0.01). We illustrated the interaction pattern in Figure 2, and the results of the simple slope test show that the positive relationship between procedural justice and competence was significant when abusive supervision was low (*b* = 0.27, *p* < 0.001) and not significant when it was high (*b* = 0.01, *p* = 0.84) [91]. Thus, we determined that Hypothesis 3 was supported. 

Lastly, we tested Hypothesis 4 to investigate whether abusive supervision moderated the indirect effect of procedural justice on creativity via competence. To assess the indirect effect, we applied bootstrapping with 10,000 samples and estimated the indirect effect of procedural justice on creativity via competence at both high (+1 SD) and low (−1 SD) levels of abusive supervision. As shown in Table 5, the results confirmed that the indirect effect was significant for low abusive supervision (indirect effect = 0.12, SE = 0.04, 95 % CI [0.05, 0.21]) but was not significant for high abusive supervision (indirect effect = 0.02, SE = 0.04, 95 % CI [−0.07, 0.10]). Therefore, we determined that Hypothesis 4 was supported.

## 5. Discussion

### 5.1. Theoretical Contributions

By proposing several important implications, this study theoretically contributes to resource conservation theory and creativity mechanisms. First, based on COR theory, we hypothesized and affirmed the relationships between procedural justice, competence, abusive supervision, and creativity, all of which are considered essential job resources. Previous researchers have explained creativity in the framework of resource conservation theory, but materialize the theory’s application, so it was necessary to clarify the structure and relationships of related variables. In our doing so, we contribute to expanding the theory and the in-depth knowledge about the relationship between variables and creativity.

Second, this study revealed the mediating role of competence as a mechanism for explaining the relationship between procedural justice and creativity. Individuals with a strong sense of competence choose supportive environments to achieve their work goals and respond more sensitively to external factors that aid the work process. As a result, competence can be inferred to function as an individual job resource and to play a role in influencing procedural justice, which is a critical job resource. This study is significant because we inferred and empirically confirmed the process of procedural justice increasing members’ competence, which, based on conservation of resources theory, leads to creativity

Lastly, with this study, we emphasized the role of leadership in the organizational factor effect by revealing that the impact of procedural justice can vary depending on the level of perceived abusive supervision among its members. Members of an organization obtain and hold various job resources from the organization and from their superiors and colleagues. It can be assumed that they will influence each other in terms of generating creativity, which is a variable outcome. The purpose of this study was to investigate the moderated mediating effect of abusive supervision, and we have made a theoretical contribution to the literature by empirically demonstrating that the role of leadership is critical for procedural justice and competence, which are job resources, to lead to actual creativity.

### 5.2. Managerial Implications

This study’s practical implications are as follows. First, by empirically demonstrating that procedural justice can be an antecedent factor in creativity as a job resource, we propose what kind of organizational justice should be offered to encourage employee creativity. As many scholars have argued through prior studies, creativity has a significant impact on organizational performance. Organizational justice, especially procedural justice, functions as a job resource that makes employees willing to approach high-level job demands with creativity.

Second, this study revealed a mediating role of competence as a mechanism to explain the relationship between procedural justice and creativity. This provides insight that employees’ competence is a job resource at the individual level and that other variables, including leadership styles, can play an important role in activating creativity. This provides an appropriate direction for organizations intending to manage antecedent factors in with regard to employee creativity.

Lastly, in this study, high levels of abusive supervision led to an insignificant indirect effect of procedural justice on the creativity of members through competence. In other words, rather than a vague expectation that the procedural justice effect will activate for employees, organizations must implement human resource management with a realistic perspective that the procedural justice effect can differ depending on the employees’ recognition of the type of leadership [92].

### 5.3. Limitations and Future Research Directions

Although this study provides meaningful implications for both scholars and practitioners, future researchers should consider some limitations. First, although we used research data obtained through a second survey with a time lag, the study has limitations as a cross-sectional study because the measurement of each study variable was limited to each particular time point. Given the time-dependent relationships among variables, future researchers should attempt a longitudinal study that could more effectively establish a causal relationship.

Second, because this study is based on data from employees from South Korean companies only, it is possible that the cultural background significantly influenced the employees’ perceptions and attitudes. Therefore, we must be cautious when interpreting and applying our results to employees in other cultural environments.

Third, because we measured all the research variables from the same source, the study is not free from concerns about common method bias. Although we staggered the survey administration to separate response time points, fundamental limitations exist. As a result of the CFA, the research models’ variables were distinguished in this study, but future researchers should consider this issue.

Fourth, in this study, we used psychological empowerment and motivational competencies that fit the purpose of the study. However, we think that examining the competencies of various frames and conducting research in more detail can provide meaningful implications. Also, if we think of abusive supervision as leadership from a situational perspective, except for the emotional abuse part, some parts are similar to the leader’s behavior in the low-maturity stage of the subordinates. In other words, abusive supervision can also be treated as a form of situational leadership in a subordinate’s maturity stage or in an urgent or exploratory setting. For example, prior research suggested a curvilinear relationship between abusive supervision and creativity. In other words, employees were most creative when they were at intermediate levels of abusive supervision than when they had high or low levels of abusive supervision [93]. In this respect, it is meaningful to examine abusive supervision with situational leadership, even though, in this study, based on resource conservation theory, we verified the interaction effect of recognizing procedural justice and abusive supervision as a more comprehensive job resource for employees’ sense of competence. However, we think that studying impersonal supervision, competence, and creativity in a more subdivided manner according to maturity stages in the framework of situational leadership theory can also provide meaningful implications.

Fifth, we considered job resources to be the major variables based on conservation of resources theory and demonstrated their influence on creativity. In subsequent research, it is necessary to review the following research directions. From the theoretical perspective, we examined the relationships between the study variables and their implications in the context of COR theory to explain creativity, and in so doing provided new insights and perspectives on the application and extension of the theory.

Lastly, we confirmed abusive supervision as a major moderating variable. Other types of leadership, such as sympathetic, could also influence the impacts on creativity of other job resources such as procedural justice and competence, but we did not address this possibility. Additionally, examining the moderating function of various individual difference variables will also show meaningful results. Therefore, further research is needed to develop more detailed models that consider different moderating variables.

## 6. Conclusions

Based on the conservation of resources theory, we empirically examined how procedural justice influences creativity. The study findings confirmed the role of competence as a moderator and demonstrated that abusive supervision is an important moderating variable in the overall influence process. Primarily, when the level of abusive supervision was high, the indirect effect of procedural justice on creativity through competence was not significant. In other words, effectively inducing creativity by accumulating employee job resources, systems, and policies requires ensuring the availability of sufficient workplace resources to support employees and ensuring supportive relationships between superiors and subordinates. Despite the limitations of the study discussed above, this finding has important implications for a variety of organizations that need to encourage and activate their employees’ creativity.

## Figures and Tables

**Table 1:** Means, Standard Deviations, Correlations, and Reliabilities.

Variables	Mean	SD	1	2	3	4	5	6	7	8	9
1. Gender	0.53	0.50	-								
2. Age	41.81	10.34	0.02	-							
3. Education	2.79	1.07	0.07	−0.04	-						
4. Job level	2.59	1.54	0.37 ***	0.49 ***	0.22 ***	-					
5. Tenure	7.98	7.47	0.16 **	0.51 ***	0.10	0.43 ***	-				
6. JTC	3.14	0.68	0.01	0.11 *	0.01	0.19 ***	0.19 ***	(0.79)			
7. ASV	2.04	0.83	0.03	0.02	0.01	−0.05	−0.06	−0.22 ***	(0.94)		
8. CPT	3.67	0.61	0.01	0.08	0.10	0.11 *	0.08	0.22 ***	−0.18 **	(0.85)	
9. CRV	3.30	0.68	0.03	0.11 *	0.12 *	0.18 ***	0.10	0.22 ***	0.00	0.47 ***	(0.87)

Notes: *n* = 337, * *p* < 0.05, ** *p* < 0.01, *** *p* < 0.001, the values in parentheses denote Cronbach’s alphas. Age: years, Gender: female = 0, male = 1, Education = highest education level achieved: 1 = high school graduates, 2 = college graduates, 3 = university graduates, 4 = post-graduates, 5 = Ph.D. holders. Job level: 1 = staff, 2 = assistant manager, 3 = manager, 4 = senior manager, 5 = directors, 6 = executives. Tenure: organizational tenure (year), JTC = procedural justice, ASV = abusive supervision, CPT = competence, CRV = creativity.

**Table 2:** Confirmatory Factor Analysis Results.

Model.	χ^2^ (df)	CFI	TLI	RMSEA	Δχ^2^ (Δdf) ^4^
Research model (4 factor)	216.78(158) **	0.98	0.98	0.03	
Alternative model 1 (3 factor) ^1^	641.98(166) ***	0.85	0.82	0.09	424.80(8) ***
Alternative model 2 (2 factor) ^2^	1152.30(173) ***	0.70	0.65	0.13	933.52(15) ***
Alternative model 3 (1 factor) ^3^	1836.97(179) ***	0.48	0.42	0.17	1620.19(21) ***

**Notes:***n* = 377, ** *p* < 0.01 *** *p* <0.001, CFI = comparative fit index, TLI = Tucker–Lewis index, RMSEA = root mean square; ^1^ 3 factor: JTC + ASV, CPT, CRV, ^2^ 2 factor: JTC + ASV+CPT, CRV, ^3^ 1 factor: JTC + ASV + CPT + CRV, JTC = procedural justice, ASV = abusive supervision, CPT = competence, CRV = creativity, ^4^ Chi-square difference for each model reflects its deviation from the four-factor model.

**Table 3:** Hierarchical Multiple Regression Results.

Variables	CPT			CRV		
	Model 1	Model 2	Model 3	Model 4	Model 5	Model 6
Gender	−0.03	−0.01	−0.01	−0.03	−0.01	−0.02
Age	0.04	0.05	0.06	0.04	0.05	0.02
Education	0.09	0.10	0.12 *	0.09	0.10	0.05
Job level	0.08	0.04	0.03	0.15 *	0.11	0.10
Tenure	0.02	−0.01	−0.02	0.01	−0.02	−0.01
JTC		0.20 ***	0.15 **		0.20 ***	0.14 **
ASV			−0.17 **			0.12 *
JTC * ASV			−0.14 *			
CPT						0.44 ***
*R* ^2^	0.02	0.06	0.10	0.04	0.08	0.27
Δ*R*^2^		0.04	0.04		0.04	0.19
*R*^2^_a	0.01	0.04	0.08	0.03	0.06	0.25
*F*	1.51	3.60 **	4.48 ***	2.95 *	4.87 ***	14.77 ***
*F*_inc_		13.75 ***	6.73 **	6.76 ***	13.90 ***	40.96 ***

Note. *n* = 337, * *p* < 0.05, ** *p* < 0.01, *** *p* < 0.001 (two-tailed test), Standardized coefficients are reported, JTC=procedural justice, ASV=abusive supervision, CPT = competence, CRV = creativity.

**Table 4:** Bootstrapping Mediation Results.

	Dependent Variable: CRV			
Mediator	Indirect Effect	SE	95% CI	
			LL	UL
CPT	0.10	0.03	0.04	0.15

Note. *n* = 337, Bootstrap sample size = 10,000, CPT = competence, CRV = creativity, SE = standard error, CI = confidence interval, LL = lower limit, UL = upper limit.

**Table 5:** Moderated Mediation Bootstrapping Results.

		Dependent Variable: CRV			
Moderator	Level	Indirect Effect	SE	95% CI	
				LL	UL
ASV	Low (−1 SD)	0.12	0.04	0.05	0.21
	High (+1 SD)	0.02	0.04	−0.07	0.10

Notes: *n* = 337, bootstrap sample size = 10,000, ASV = abusive supervision, CRV = creativity, SE = standard error, CI = confidence interval, LL = lower limit, UL = upper limit.

**Figure A1:**
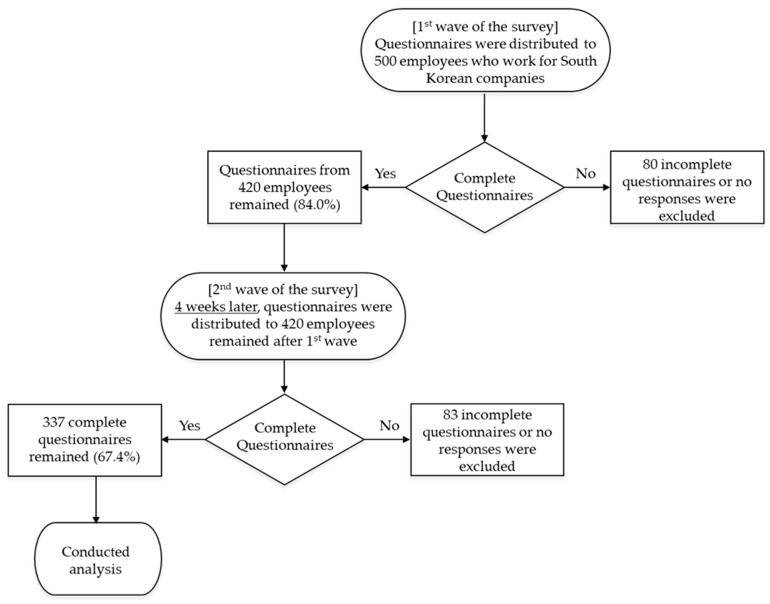
Sampling Procedure.

## Appendix B. Measurements

Procedural justice (Cronbach’s alpha = 0.79) [79].

1. People involved in implementing decisions have a say in making the decisions.
2. Members of my work unit are involved in making decisions that directly affect their work.
3. Decisions are made on the basis of research, data, and technical criteria, as opposed to political concerns.
4. People with the most knowledge are involved in the resolution of problems.

Abusive supervision (Cronbach’s alpha = 0.94) [80].

1. Ridicules me.
2. Tells my thoughts or feelings are stupid.
3. Puts me down in front of others.
4. Makes negative comments about me to others.
5. Tells me I’m incompetent.

Competence (Cronbach’s alpha = 0.85) [49].

1. I am confident about my ability to do my job.
2. I am self-assured about my capabilities to perform my work activities.
3. I have mastered the skills necessary for my job.

Creativity (Cronbach’s alpha = 0.87) [81].

1. I supplied new ideas and differing perspectives.
2. I raised interesting issues and challenging questions for discussion.
3. I actively listened to others and integrated their ideas to offer creative solutions.
4. I combined ideas from different modules and came up with a more integrated view of the phenomena.

## References

[1] Grant A.M., Ashford S.J. (2008). The dynamics of proactivity at work. Res. Organ. Behav..

[2] Amabile T.M., Pillemer J. (2012). Perspectives on the Social Psychology of Creativity. J. Creat. Behav..

[3] Yuan F., Woodman R.W. (2010). Innovative Behavior in the Workplace: The Role of Performance and Image Outcome Expectations. Acad. Manag. J..

[4] Oldham G.R., Cummings A. (1996). Employee Creativity: Personal and Contextual Factors at Work. Acad. Manag. J..

[5] Scott S.G., Bruce R.A. (1994). Determinants of Innovative Behavior: A Path Model of Individual Innovation in the Workplace. Acad. Manag. J..

[6] Jeong S.-E., Choi B.-W., Chung T.-Y. (2018). The Foundation of Business Administration.

[7] Amabile T.M. (1988). A Model of Creativity and Innovation in Organizations. Res. Organ. Behav..

[8] Jo S.-J. (2019). History of Business and Management.

[9] Malik N., Tripathi S.N., Kar A.K., Gupta S. (2021). Impact of artificial intelligence on employees working in industry 4.0 led organizations. Int. J. Manpow..

[10] Locke R., Castrucci B.C., Gambatese M., Sellers K., Fraser M. (2019). Unleashing the Creativity and Innovation of Our Greatest Resource-The Governmental Public Health Workforce. J. Public Health Manag. Pract. JPHMP.

[11] Chang L.-C., Liu C.-H. (2008). Employee empowerment, innovative behavior and job productivity of public health nurses: A cross-sectional questionnaire survey. Int. J. Nurs. Stud..

[12] García-Goñi M., Maroto A., Rubalcaba L. (2007). Innovation and motivation in public health professionals. Health Policy.

[13] Amabile T.M. (1983). The social psychology of creativity: A componential conceptualization. J. Personal. Soc. Psychol..

[14] Amabile T.M. (1997). Motivating Creativity in Organizations: On Doing What You Love and Loving What You Do. Calif. Manag. Rev..

[15] Shalley C.E., Zhou J., Oldham G.R. (2004). The Effects of Personal and Contextual Characteristics on Creativity: Where Should We Go from Here?. J. Manag..

[16] Amabile T.M., Conti R., Coon H., Lazenby J., Herron M. (1996). Assessing the Work Environment for Creativity. Acad. Manag. J..

[17] Deci E.L., Ryan R.M. (1980). Self-determination Theory: When Mind Mediates Behavior. J. Mind Behav..

[18] Howell J.M., Higgins C.A. (1990). Champions of change: Identifying, understanding, and supporting champions of technological innovations. Organ. Dyn..

[19] Nonaka I. (1994). A Dynamic Theory of Organizational Knowledge Creation. Organ. Sci..

[20] Marion R., Mumford M.D. (2012). Chapter 18—Leadership of Creativity: Entity-Based, Relational, and Complexity Perspectives. Handbook of Organizational Creativity.

[21] Anderson N., Potočnik K., Zhou J. (2014). Innovation and Creativity in Organizations:A State-of-the-Science Review, Prospective Commentary, and Guiding Framework. J. Manag..

[22] Sung S.Y., Rhee Y.W., Lee J.E., Choi J.N. (2020). Dual pathways of emotional competence towards incremental and radical creativity: Resource caravans through feedback-seeking frequency and breadth. Eur. J. Work Organ. Psychol..

[23] Ghafoor A., Haar J. (2020). A Climate and Personality Approach towards Creativity behaviours: A moderated mediation study. Int. J. Innov. Manag..

[24] Du J., Ma E., Cabrera V., Jiao M. (2021). Keep your mood up: A multilevel investigation of hospitality employees’ positive affect and individual creativity. J. Hosp. Tour. Manag..

[25] Akinola M., Kapadia C., Lu J.G., Mason M.F. (2019). Incorporating Physiology into Creativity Research and Practice: The Effects of Bodily Stress Responses on Creativity in Organizations. Acad. Manag. Perspect..

[26] Bakker A.B., Demerouti E. (2017). Job demands-resources theory: Taking stock and looking forward. J. Occup. Health Psychol..

[27] Boudrias J.S., Desrumaux P., Gaudreau P., Nelson K., Brunet L., Savoie A. (2011). Modeling the experience of psychological health at work: The role of personal resources, social-organizational resources, and job demands. Int. J. Stress Manag..

[28] Amabile T.M., Conti R. (1999). Changes in the Work Environment for Creativity During Downsizing. Acad. Manag. J..

[29] Amabile T.M., Gryskiewicz N.D. (1989). The creative environment scales: Work environment inventory. Creat. Res. J..

[30] Shalley C.E., Gilson L.L. (2004). What leaders need to know: A review of social and contextual factors that can foster or hinder creativity. Leadersh. Q..

[31] Tepper B.J. (2000). Consequences of Abusive Supervision. Acad. Manag. J..

[32] Tepper B.J., Moss S.E., Lockhart D.E., Carr J.C. (2007). Abusive Supervision, Upward Maintenance Communication, and Subordinates’ Psychological Distress. Acad. Manag. J..

[33] Barnes C.M., Lucianetti L., Bhave D.P., Christian M.S. (2015). “You Wouldn’t Like Me When I’m Sleepy”: Leaders’ Sleep, Daily Abusive Supervision, and Work Unit Engagement. Acad. Manag. J..

[34] Truxillo D.M., Bauer T.N., Sanchez R.J. (2001). Multiple Dimensions of Procedural Justice: Longitudinal Effects on Selection System Fairness and Test-Taking Self-Efficacy. Int. J. Sel. Assess..

[35] Zhang Y., Long L., Zhang J. (2015). Pay for performance and employee creativity. Manag. Decis..

[36] Hobfoll S.E. (2001). The Influence of Culture, Community, and the Nested-Self in the Stress Process: Advancing Conservation of Resources Theory. Appl. Psychol..

[37] Hobfoll S.E. (1989). Conservation of resources. A new attempt at conceptualizing stress. Am. Psychol..

[38] Cropanzano R., Byrne Z.S., Bobocel D.R., Rupp D.E. (2001). Moral Virtues, Fairness Heuristics, Social Entities, and Other Denizens of Organizational Justice. J. Vocat. Behav..

[39] Leventhal G.S., Greenberg M.S., Gergen K.J., Willis R.H. (1980). What Should Be Done with Equity Theory?. Social Exchange.

[40] Thibaut J., Walker L. (1975). Procedural Justice: A Psychological Analysis.

[41] Cropanzano R., Bowen D.E., Gilliland S.W. (2007). The Management of Organizational Justice. Acad. Manag. Perspect..

[42] Moorman R.H. (1991). Relationship between organizational justice and organizational citizenship behaviors: Do fairness perceptions influence employee citizenship?. J. Appl. Psychol..

[43] Organ D.W. (1988). Organizational Citizenship Behavior: The Good Soldier Syndrome.

[44] McFarlin D.B., Sweeney P.D. (1992). Research Notes. Distributive and Procedural Justice as Predictors of Satisfaction with Personal and Organizational Outcomes. Acad. Manag. J..

[45] Folger R., Konovsky M.A. (1989). Effects of Procedural and Distributive Justice on Reactions to Pay Raise Decisions. Acad. Manag. J..

[46] Mehmood S.A., Malik M.A.R., Akhtar M.S., Faraz N.A., Memon M.A. (2021). Organizational justice, psychological ownership and organizational embeddedness: A conservation of resources perspective. Int. J. Manpow..

[47] De Clercq D., Ul Haq I., Azeem M.U. (2021). Unpacking the relationship between procedural justice and job performance. Manag. Decis..

[48] De Clercq D., Pereira R. (2020). Knowledge-sharing efforts and employee creative behavior: The invigorating roles of passion for work, time sufficiency and procedural justice. J. Knowl. Manag..

[49] Spreitzer G.M. (1995). Psychological Empowerment in the Workplace: Dimensions, Measurement, and Validation. Acad. Manag. J..

[50] McClelland D.C. (1973). Testing for competence rather than for “intelligence”. Am. Psychol..

[51] Thomas K.W., Velthouse B.A. (1990). Cognitive Elements of Empowerment: An “Interpretive” Model of Intrinsic Task Motivation. Acad.Manag. Rev..

[52] Deci E.L., Ryan R.M. (1985). Intrinsic Motivation and Self-Determination in Human Behavior.

[53] Deci E.L., Ryan R.M. (2002). Self-determination research: Reflections and future directions. Handbook of Self-Determination Research.

[54] Ryan R.M., Deci E.L. (2000). Intrinsic and Extrinsic Motivations: Classic Definitions and New Directions. Contemp. Educ. Psychol..

[55] Bandura A., Watts R.E. (1996). Self-Efficacy in Changing Societies. J. Cogn. Psychother..

[56] Guay F., Senécal C., Gauthier L., Fernet C. (2003). Predicting career indecision: A self-determination theory perspective. J. Couns. Psychol..

[57] Bandura A. (1977). Self-efficacy: Toward a unifying theory of behavioral change. Psychol. Rev..

[58] Stajkovic A.D., Luthans F. (1998). Social cognitive theory and self-efficacy: Goin beyond traditional motivational and behavioral approaches. Organ. Dyn..

[59] Sherer M., Maddux J.E., Mercandante B., Prentice-Dunn S., Jacobs B., Rogers R.W. (1982). The Self-Efficacy Scale: Construction and Validation. Psychol. Rep..

[60] Schmidt A.M., DeShon R. (2010). The moderating effects of performance ambiguity on the relationship between self-efficacy and performance. J. Appl. Psychol..

[61] Zimmerman B.J. (2000). Self-Efficacy: An Essential Motive to Learn. Contemp. Educ. Psychol..

[62] Breevaart K., Bakker A.B., Demerouti E., Derks D. (2016). Who takes the lead? A multi-source diary study on leadership, work engagement, and job performance. J. Organ. Behav..

[63] Newman A., Herman H.M., Schwarz G., Nielsen I. (2018). The effects of employees’ creative self-efficacy on innovative behavior: The role of entrepreneurial leadershi. J. Bus. Res..

[64] Fiernaningsih N., Pudji Herijanto M. (2021). Antecedents of variables that affect innovative behavior in the era of the COVID-19 pandemic. PalArch’s J. Archaeol. Egypt/Egyptol..

[65] Zheng M.X., Schuh S.C., van Dijke M., De Cremer D. (2021). Procedural justice enactment as an instrument of position protection: The three-way interaction between leaders’ power position stability, followers’ warmth, and followers’ competence. J. Organ. Behav..

[66] Zhang Y., Liao Z. (2015). Consequences of abusive supervision: A meta-analytic review. Asia Pac. J. Manag..

[67] Bamberger P.A., Bacharach S.B. (2006). Abusive supervision and subordinate problem drinking: Taking resistance, stress and subordinate personality into account. Human Relat..

[68] Dupré K.E., Inness M., Connelly C.E., Barling J., Hoption C. (2006). Workplace aggression in teenage part-time employees. J. Appl. Psychol..

[69] Schyns B., Schilling J. (2013). How bad are the effects of bad leaders? A meta-analysis of destructive leadership and its outcomes. Leadersh. Q..

[70] Hobfoll S.E., Freedy J., Lane C., Geller P. (1990). Conservation of Social Resources: Social Support Resource Theory. J. Soc. Pers. Relatsh..

[71] Thomas G., Martin R., Epitropaki O., Guillaume Y., Lee A. (2013). Social cognition in leader–follower relationships: Applying insights from relationship science to understanding relationship-based approaches to leadershi. J. Organ. Behav..

[72] Lee H.-M., Ko Y.-J., Kim G.-R., Song Y.-S. (2020). A Study on the Effects of Intrinsic Motivation and Creative Process Engagement on Organizational Creativity: Abusive Supervision of the Superior in the Organization as Moderator. J. Corp. Educ. Talent Res..

[73] Aryee S., Walumbwa F.O., Zhou Q., Hartnell C.A. (2012). Transformational Leadership, Innovative Behavior, and Task Performance: Test of Mediation and Moderation Processes. Hum. Perform..

[74] Zellars K.L., Tepper B.J., Duffy M.K. (2002). Abusive supervision and subordinates’ organizational citizenship behavior. J. Appl. Psychol..

[75] Podsakoff P.M., MacKenzie S.B., Lee J.Y., Podsakoff N.P. (2003). Common method biases in behavioral research: A critical review of the literature and recommended remedies. J. Appl. Psychol..

[76] Dawson K., O’Brien K., Beehr T. (2015). The role of hindrance stressors in the job demand–control–support model of occupational stress: A proposed theory revision. J. Organ. Behav..

[77] Daniels K., Guppy A. (1994). Occupational Stress, Social Support, Job Control, and Psychological Well-Being. Hum. Perform..

[78] Brislin R.W., Altman I., Rapoport A., Wohlwill J.F. (1980). Cross-Cultural Research Methods. Environment and Culture.

[79] Parker C.P., Baltes B.B., Christiansen N.D. (1997). Support for affirmative action, justice perceptions, and work attitudes: A study of gender and racial–ethnic group differences. J. Appl. Psychol..

[80] Mitchell M.S., Ambrose M.L. (2007). Abusive supervision and workplace deviance and the moderating effects of negative reciprocity beliefs. J. Appl. Psychol..

[81] Sung S.Y., Choi J.N. (2009). Do Big Five personality factors affect individual creativity? The moderating role of extrinsic motivation. Soc. Behav. Personal. Int. J..

[82] Wright T., Cropanzano R. (2000). Psychological well-being and job satisfaction as predictors of job performance. J. Occup. Health Psychol..

[83] Preacher K.J., Hayes A.F. (2008). Asymptotic and resampling strategies for assessing and comparing indirect effects in multiple mediator models. Behav. Res. Methods.

[84] Hayes A.F. (2018). Partial, conditional, and moderated moderated mediation: Quantification, inference, and interpretation. Commun. Monogr..

[85] Hair J.F., Black W.C., Babin B.J., Anderson R.E. (2010). Multivariate Data Analysis: A Global Perspective.

[86] Fornell C., Larcker D.F. (1981). Structural Equation Models with Unobservable Variables and Measurement Error: Algebra and Statistics. J. Mark. Res..

[87] Asyraf W.M., Afthanorhan B.W. (2013). A Comparison Of Partial Least Square Structural Equation Modeling (PLS-SEM) and Covariance Based Structural Equation Modeling (CB-SEM) for Confirmatory Factor Analysis. Int. J. Eng. Sci. Innov. Technol..

[88] Fornell C., Larcker D.F. (1981). Evaluating structural equation models with unobservable variables and measurement error. J. Mark. Res..

[89] Bagozzi R., Yi Y. (1988). On the evaluation of structural equation models. J. Acad. Mark. Sci..

[90] Fuller C.M., Simmering M.J., Atinc G., Atinc Y., Babin B.J. (2016). Common methods variance detection in business research. J. Bus. Res..

[91] Aiken L.S., West S.G. (1991). Multiple Regression: Testing and Interpreting Interactions.

[92] Jo S.-J., Bae E.G., Kim H.S., Kim D.Y., Lee M.Y., Rhee S.S., Choi W.J. (2018). Models for HRD Practice: Career Development.

[93] Lee S., Yun S., Srivastava A. (2013). Evidence for a curvilinear relationship between abusive supervision and creativity in South Korea. Leadersh. Q..

